# Supplementary material for: CaV2.1 channel mutations causing familial hemiplegic migraine type 1 increase the susceptibility for cortical spreading depolarizations and seizures and worsen outcome after experimental traumatic brain injury
Source: eLife. 2022 Mar 3;11:e74923. doi: 10.7554/eLife.74923 (PMC8920504; doi:10.7554/eLife.74923)
Supplement: Transparent reporting form [file elife-74923-transrepform1.pdf]

eLIFE
1st Floor
24 Hills Road
Cambridge CB2 1JP, UK
P 01223 855340
W elifesciences.org
T @elife

# *eLife's* transparent reporting form

We encourage authors to provide detailed information *within their submission* to facilitate the interpretation and replication of experiments. Authors can upload supporting documentation to indicate the use of appropriate reporting guidelines for health-related research (see EQUATOR Network), life science research (see the BioSharing Information Resource), or the ARRIVE guidelines for reporting work involving animal research. Where applicable, authors should refer to any relevant reporting standards documents in this form.

If you have any questions, please consult our Journal Policies and/or contact us: editorial@elifesciences.org.

**Sample-size estimation**

- You should state whether an appropriate sample size was computed when the study was being designed
- You should state the statistical method of sample size computation and any required assumptions
- If no explicit power analysis was used, you should describe how you decided what sample (replicate) size (number) to use

Please outline where this information can be found within the submission (e.g., sections or figure legends), or explain why this information doesn't apply to your submission:

All animal experiments performed at our Institution have to undergo a rigorous review by the Animal Ethics Committee of the Veterinary Office of the Government of Upper Bavaria. This review includes a detailed evaluation of sample size calculation by a professional statistician. This is information is provided at the **beginning of the Material and Methods section**.

**Replicates**

- You should report how often each experiment was performed
- You should include a definition of biological versus technical replication
- The data obtained should be provided and sufficient information should be provided to indicate the number of independent biological and/or technical replicates
- If you encountered any outliers, you should describe how these were handled
- Criteria for exclusion/inclusion of data should be clearly stated
- High-throughput sequence data should be uploaded before submission, with a private link for reviewers provided (these are available from both GEO and ArrayExpress)

Please outline where this information can be found within the submission (e.g., sections or figure legends), or explain why this information doesn't apply to your submission:

The **size of each experimental group** is provided within the **Results section and within each figure**.
The information that we **did not exclude any data** is provided at the **beginning of the Material and Methods section**.

**Statistical reporting**

- Statistical analysis methods should be described and justified

eLife Sciences Publications, Ltd is a limited liability non-profit non-stock corporation incorporated in the State of Delaware, USA, with company number 5030732, and is registered in the UK with company number FC030576 and branch number BR015634 at the address 1st Floor, 24 Hills Road, Cambridge CB2 1JP | August 2014

- Raw data should be presented in figures whenever informative to do so (typically when N per group is less than 10)
- For each experiment, you should identify the statistical tests used, exact values of N, definitions of center, methods of multiple test correction, and dispersion and precision measures (e.g., mean, median, SD, SEM, confidence intervals; and, for the major substantive results, a measure of effect size (e.g., Pearson's r, Cohen's d)
- Report exact p-values wherever possible alongside the summary statistics and 95% confidence intervals. These should be reported for all key questions and not only when the p-value is less than 0.05.

Please outline where this information can be found within the submission (e.g., sections or figure legends), or explain why this information doesn't apply to your submission:

The **statistical approach** is described in detail at the **end of the Material and Methods section**. The **individual statistics** of each graph are given in the **figure legend**.
**All data** are presented as bar graphs (**median ± 95% confidence interval** overlaid with scatter plots representing **individual values**). A separate file containing a spreadsheet with all **raw data** from each individual animal **is provided** to allow replication of our statistical approach.

(For large datasets, or papers with a very large number of statistical tests, you may upload a single table file with tests, Ns, etc., with reference to sections in the manuscript.)

**Group allocation**

- Indicate how samples were allocated into experimental groups (in the case of clinical studies, please specify allocation to treatment method); if randomization was used, please also state if restricted randomization was applied
- Indicate if masking was used during group allocation, data collection and/or data analysis

Please outline where this information can be found within the submission (e.g., sections or figure legends), or explain why this information doesn't apply to your submission:

Experiments were performed in a **randomized** manner by **drawing lots** and researchers were **blinded** towards the genotype of the animals **until the end of data analysis**. This information is provided at the **Material and Method section**, **subheading "Experimental design"**.

**Additional data files ("source data")**

- We encourage you to upload relevant additional data files, such as numerical data that are represented as a graph in a figure, or as a summary table
- Where provided, these should be in the most useful format, and they can be uploaded as "Source data" files linked to a main figure or table
- Include model definition files including the full list of parameters used
- Include code used for data analysis (e.g., R, MatLab)
- Avoid stating that data files are "available upon request"

Please indicate the figures or tables for which source data files have been provided:

Two Excel files containing all raw data presented in the manuscript have been uploaded to the Center for Open Science (OSF) under the following link:
https://osf.io/jqae2/?view_only=e8bbad9eadce435b8b781b4a781e1248
This link is also provided in the Material & Methods part of our manuscript.
